# Optimal distribution of the fundamental non-efficient load current terms between the energy gateways connected in grid-tied microgrids

**DOI:** 10.1016/j.heliyon.2023.e13650

**Published:** 2023-02-13

**Authors:** Santiago Benavides-Córdoba, Jorge-Humberto Urrea-Quintero, Nicolás Muñoz-Galeano, Juan-B Cano-Quintero, Salvador Segui-Chilet

**Affiliations:** a Universidad de Antioquia, Grupo de Investigación GIMEL, Departamento de Ingeniería Eléctrica, calle 67 number 53-108, Medellín, 050010, Antioquia, Colombia; b Instituto Interuniversitario de Investigación de Reconocimiento Molecular y Desarrollo Tecnológico (IDM), Universitat Politècnica de València, Camino de Vera 14, Valencia, 46022, Valencia, Spain

**Keywords:** Microgrid, Power quality, Energy gateways, Non-efficient load current optimal distribution, Current sharing

## Abstract

Main objective of this paper is the optimal distribution of the fundamental non-efficient load current terms between the inverters —Energy Gateways (EGs)— connected in grid-tied microgrids (MGs). The main feature of the presented approach is the use of the EGs as controlled current sources that can compensate fundamental non-efficient load current terms in addition to the generation of fundamental positive-sequence active current, avoiding the use of shunt active power filters. The approach relies on the so-called System of Constant References (SoCR) that is based on the symmetrical components decomposition and *dq0* transformation. SoCR procedure decouples efficient and non-efficient components of the instantaneous load currents, transforming all of them into six constant references. The optimization algorithm uses a new approach for the calculation of the peak currents in each phase, avoiding non-convex problems when determining the currents of the EGs considering their operating limits. A medium-power size MG that includes photovoltaic and wind generators, as well as, a battery energy storage system is considered to evaluate the capabilities of the proposal. There were evaluated four scenarios: baseline, balanced distribution, proportional distribution, and optimal distribution. All scenarios, except optimal distribution scenario, surpass the current limits for the EGs connected. The results highlight the benefits of using the EGs as active agents in MG efficient operation and demonstrate how the optimization approach achieves the goal of maintaining the generation capabilities of EGs at the same time that compensates the non-efficient current terms demanded by the load.

## Highlights

• A method to distribute the tasks of an active filter among the energy gates of the micro-grid is presented.
• An optimization model is proposed for the distribution of non-efficient current terms between the EGs in a microgrid.
• The positive sequence current term is decoupled from their corresponding non-efficient fundamental current terms.
• A system of constant references (SoCR) is proposed to represent an unbalanced three-phase linear system.
• The decoupling achieved with SoCR allows distributing the non-efficient terms among the EGs of a grid-tied MG.

## Graphical abstract

**Figure:**
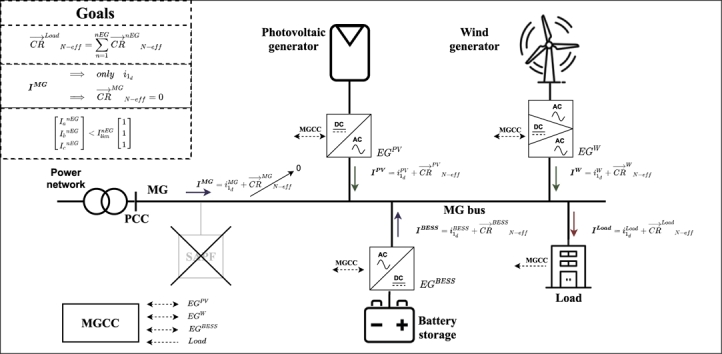

## 1 Introduction

Microgrids (MGs) based on renewable energy sources have been described as the base brick of modern electric systems [1], [2]. Their adoption is an alternative to achieve high standards of power quality and efficiency in distribution power networks, while satisfying the growing energy demand. However, new challenges have emerged due to the intrinsic intermittency of renewable energy sources, leading in some cases to a deterioration of the electrical grid reliability or even causing some instability problems [3], [4]. Hence, MG optimal operation while minimizing its side effects is still a topic of active research [4], [5].

An important issue in MGs is the problem related to fundamental unbalance and reactive power demand [2], [6], [7]. For instance, in a grid-tied MG the problems related to non-efficient power terms are transferred to the power network and the following two problems could arise: 1) reactive power is transferred to the distribution network, therefore, the power network capacity for the feeding of active power decreases; and 2) unbalance could limit the power availability in one or two MG branches due to a phase or phases overloading [6], [8]. Additionally, other loads connected to the power network could be affected by these non-efficient powers. There are several problems related to the unbalance in electrical machines, namely, vibrations, overheating, and torque loss. For these reasons, power quality issues are among the most challenging topics of controlling an MG [6], [9].

In grid-tied MGs, unbalance and reactive powers have been traditionally compensated by means of Shunt Active Power Filters (SAPFs), where the SAPF supplies all the non-efficient power terms demanded by the loads in the power line in which the SAPF is connected. A review concerning unbalance and reactive power compensation strategies was presented in [10]. Even though installing an SAPF is a good option to compensate the non-efficient power terms in grid-tied MGs, as stated in [11], [12], [13], adopting SAPFs increase the total cost of the MG because it requires an additional device connected in the MG.

In order to reduce the cost of grid-tied MGs, researchers and developers have shifted their focus on an active utilization of grid-tied energy gates (EGs) due to their flexibility [6], [10]. Basically, the existing EGs distributed along the MG could compensate the non-efficient powers of the load by avoiding their flow to the power network, with [14], [15], [16] showing the trend to include ancillary services in EGs. In general terms, EGs in MGs have the possibility to play the same role as SAPFs, enhancing electric power quality and increasing the global efficiency of the system [17].

Evidence of the relative success of the EGs application as active agents in the non-efficient powers compensation can be found in the literature. Accordingly, developments for photovoltaic (PV) [18], [19], wind [20], and battery energy storage system (BESS) combined with ancillary services for power quality improvement [21], [22] have been presented. Although these papers report some improvements in power quality, the approach followed by most of them is to compensate all non-efficient powers in the MG from a single EG, without considering current or power limits in its output. With this method, the EG should be of higher power and is therefore equivalent to including an SAPF in the MG. It is well known that the requirement of higher capacity in an EG increases its cost. Moreover, using only one EG would miss the opportunity to better utilize the total apparent power capacity distributed in the MG. In contrast, this paper proposes an optimization-based approach for the distribution of the non-efficient current terms between the EGs of the MG exploiting better their versatility as active power filters. The apparent power limits of each EG are incorporated as constraints in the optimization problem without affecting the active power that each EG can generate from renewable sources. In this way, power quality issues in MGs are mitigated without requiring EGs of higher capacity.

The distribution among the EGs of a MG of the non-efficient load currents that cause the existence of unbalance and reactive power can be performed using different optimization models, as shown in [8], [9], [23], [24], [25], [26], [27], [28]. In [24], a cooperative optimization approach without centralized supervision or additional control units was presented. MG capabilities are extended without requiring further infrastructural investments, minimizing energy losses in the distribution lines looking for full utilization of EGs by means of Token Ring Control, but neglecting power quality issues. In [25], authors proposed an optimization based on a conservative power theory, where the MG center controller (MGCC) sends commands to the EGs looking for selective elimination of the main causes affecting the power quality.

In [26], authors enhanced the system load capacity under unbalance load conditions by implementing a power routing mechanism for the EGs by means of MG supervisory control. Nonetheless, in the proposed method, active and reactive powers are coupled, which could imply a more complex signal processing. A multi-objective optimization model is proposed in [9], being focused on active power losses, voltage deviation, and the calculation of a power imbalance factor. The proposed optimization model applies to stand-alone and grid-tied MGs. However, the proposed power imbalance factor does not guarantee an unbalance compensation and the paper prioritizes the reduction of active power losses for an MG with a long distance between the EGs. There are cases where the optimization is aimed at finding a better cost function in systems with load imbalances, while trying to obtain a voltage improvement to increase the quality of power supply [23], [27]. A single-phase multi-objective optimization model for unbalance power compensation is presented for a three-phase system in [8], but the model applies only for the single-phase EGs connected in the MG.

Finally, in [28], the authors proposed a method for the distribution of the non-efficient power terms between the EGs using an instantaneous power theory, but the MGCC must handle a complex data set. This can become in a limitation if this technology wants to be adopted in commercial applications because it would require a more sophisticated hardware. The paper deals with interesting topics such as distributed and decoupled compensation of unbalance and reactive powers in low-voltage networks, fully-controllable power flow control at the point of common coupling (PCC) of the MG with the power network, coordinated control of four-leg EGs in three-phase four-wire grids, and a proportional current sharing among four-leg EGs. The author claims to be the first one that separates the active current term from the reactive and unbalance current components in the MG context considering four-leg inverters. Although, [28] implemented first a methodology to distribute non-efficient current terms between EGs, an optimization method is still needed in an MG, considering EGs nominal apparent power capacities.

As the main contribution, this paper aims to overcome most of the previous mentioned limitations by proposing an optimization model for the sharing of non-efficient fundamental load currents compensation among the connected EGs in a MG. The model considers the apparent power limits of each EG without affecting the active power that each can generate from the renewable sources, improving power quality issues in MGs. The approach is based on symmetrical components and *dq* transformation following the ideas presented by [29]. Then, an optimal decomposition of the currents is performed to obtain a System of Constant Reference (SoCR) that allows to represent the fundamental non-efficient load currents by means of six constant terms, denoted as constant references (CR). One of the SoCR terms is related to the fundamental positive-sequence active power, while the others are related to the reactive and unbalance powers that must be reduced or compensated in the MG to achieve high quality standards of power quality and efficiency.

In summary, this paper improves the previous approaches as follows:

• A general approach to exploit the versatility of the EGs is provided in such a way that the fundamental positive-sequence active power is supplied to the load and, at the same time, the non-efficient fundamental load currents are compensated in an optimal way.
• The current limits of the EG are incorporated as hard constraints in the optimization problem keeping the MG operation safe without surpassing at any time the maximum capacity of the EGs.
• Unbalance compensation is enhanced.

This paper is organized as follows. Section 2 presents the proposed SoCR approach to represent fundamental unbalanced current systems, being divided in three subsections: 2.1 elaborates on the fundamentals of SoCR; 2.2 illustrates the methodology for SoCR implementation; and 2.3 shows peak current calculation for the sharing optimization algorithm. Section 3 describes an optimization method based on SoCR for distribution of fundamental non-efficient currents among EGs in an MG. Section 4 presents the results of the SoCR implementation considering four different distribution scenarios of the non-efficient currents. First, in 4.1, how the SoCR performs the representation of unbalanced currents is presented, and the decoupling between the fundamental positive-sequence active current and the fundamental non-efficient currents is shown. Then, in 4.2 and 4.3, balanced and proportional approaches for the distribution of non-efficient currents are developed. Finally, in 4.4, results concerning the optimal approach are described. Section 5 discusses a comparison between the proposed scenarios while section 6 concludes the paper, detailing the main benefits of our proposal.

## 2 SoCR procedure for decoupling the fundamental efficient/non-efficient load current terms

**Figure 1:**
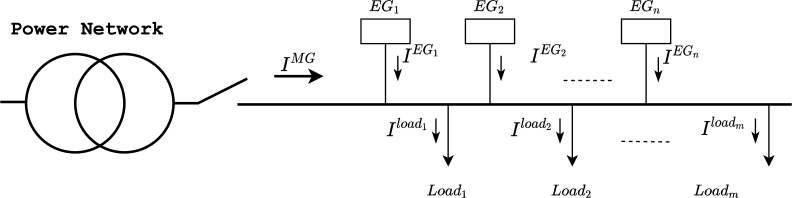
Example of a micro-grid with key elements: connection to the main grid, EGs and loads.

A generic MG is shown in Fig. 1, with *n* distributed sources of energy based on EGs and *m* different loads. This section develops a procedure for the representation of a fundamental unbalanced current system in six constant references. It starts presenting the graphical origin of the SoCR and the notation used in the paper, followed by the equations used for the implementation of the SoCR in a simulation, and it finally introduces the equation for the peak current calculation.

### 2.1 Proposed System of Constant References (SoCR)

**Figure 2:**
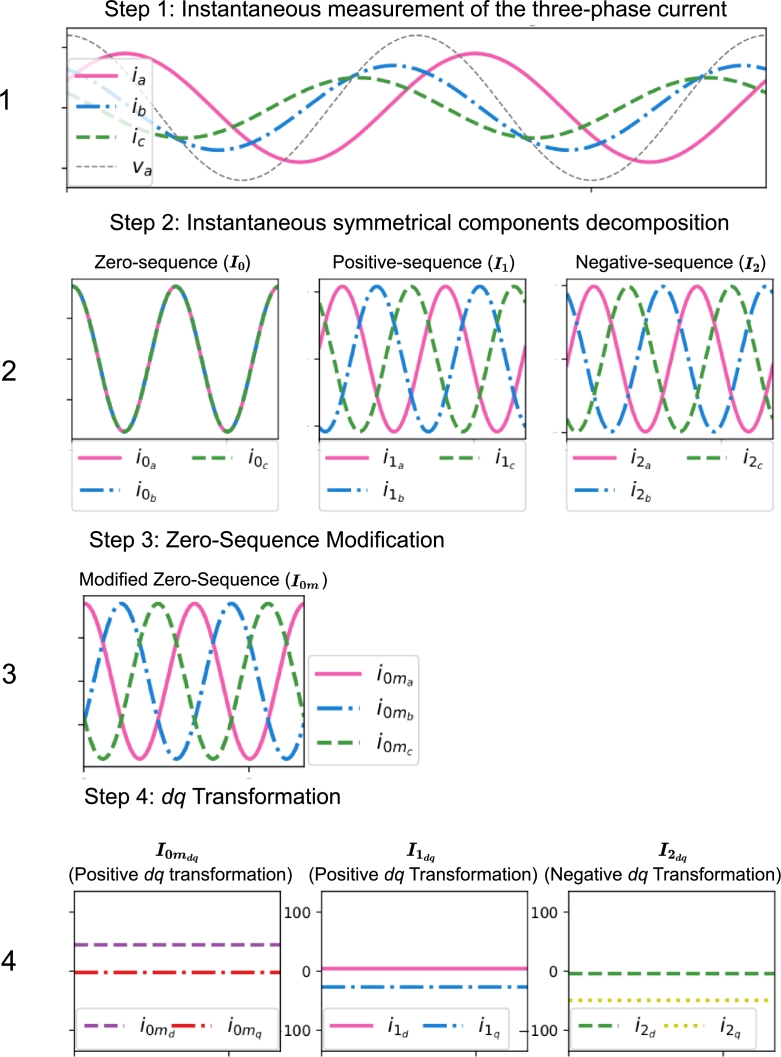
Proposed procedure for the representation of fundamental unbalanced current systems into six constant references (SoCR).

Throughout this paper, instantaneous symmetrical components decomposition methodology and *dq* transformation are used for the so-called SoCR derivation considering an MG with only fundamental positive-sequence voltages in which EGs and linear unbalanced loads are connected, without harmonic power flows [29], [30]. The SoCR is a procedure that consists of the decomposition of an instantaneous three-phase fundamental current system (top plot in Fig. 2) into six constant terms (bottom plot in Fig. 2), where one term represents the fundamental positive-sequence active current (denoted as $i_{1_{d}}$) and the rest of terms are representing all the fundamental non-efficient current terms, related to reactive and unbalance powers. This methodology can be applied to small MGs with a low inductive reactance/resistance (X/R) ratio in the line impedance, which avoids a significant voltage phase shift among the EGs [31], [28].

The three-phase currents in any point of the MG can be represented by means of a matrix representation as appears in Equation (1), where $i_{a}$, $i_{b}$, and $i_{c}$ are varying-time cosinusoidal signals. The symmetrical voltages in the MG bus have a phase shift of $\frac{2\pi }{3}$ degrees between them, forming a fundamental positive-sequence system in which phase *a* is considered the origin of the angles, being included as $v_{a}$ in the top plot in Fig. 2.

(1) $$I=\left[\begin{array}{c} i_{a}\\ i_{b}\\ i_{c} \end{array}\right]=\left[\begin{array}{c} I_{a}\cos(wt-\alpha_{a})\\ I_{b}\cos(wt-\frac{2\pi }{3}-\alpha_{b})\\ I_{c}\cos(wt-\frac{4\pi }{3}-\alpha_{c}) \end{array}\right]$$

Throughout the paper, Equation (1) is conveniently expressed considering the use of *dq* transformation (Equation (2)) as follows:

(2) $$I=\left[\begin{array}{c} I_{a_{dq}}\\ I_{b_{dq}}\\ I_{c_{dq}} \end{array}\right]=\left[\begin{array}{c} I_{a}\cos(-\alpha_{a})+jI_{a}\sin(-\alpha_{a})\\ I_{b}\cos(-\frac{2\pi }{3}-\alpha_{b})+jI_{b}\sin(-\frac{2\pi }{3}-\alpha_{b})\\ I_{c}\cos(-\frac{4\pi }{3}-\alpha_{c})+jI_{c}\sin(-\frac{4\pi }{3}-\alpha_{c}) \end{array}\right]=\left[\begin{array}{c} i_{a_{d}}+ji_{a_{q}}\\ i_{b_{d}}+ji_{b_{q}}\\ i_{c_{d}}+ji_{c_{q}} \end{array}\right]$$

The proposed SoCR decomposition is applied over the instantaneous three-phase fundamental currents measured in the EGs ($I^{nEG}$), loads ($I^{Load}$), and the MG ($I^{MG}$), following the four steps shown in Fig. 2. The first step consists of measuring ***I***, the instantaneous three-phase currents, which is decomposed into its symmetrical components, where $i_{1_{a}}$, $i_{1_{b}}$, and $i_{1_{c}}$ are the positive-sequence currents, denoted as $I_{1}$; $i_{2_{a}}$, $i_{2_{b}}$, and $i_{2_{c}}$ are the negative-sequence currents, denoted as $I_{2}$; and $i_{0_{a}}$, $i_{0_{b}}$, and $i_{0_{c}}$ are the zero-sequence currents, denoted as $I_{0}$. The third step shown in Fig. 2 generates a modified zero-sequence system, denoted as $I_{0m}$, in which a phase delay of $\frac{2\pi }{3}$ and $\frac{4\pi }{3}$ are respectively applied to *b* and *c* zero-sequence components, obtaining the terms denoted as $i_{0m_{a}}$, $i_{0m_{b}}$ and $i_{0m_{c}}$, which constitute a balanced system of positive-sequence. The fourth step consists of the application of positive or negative *dq* transformation to the corresponding sequence components, respectively. The complete SoCR procedure to obtain the six constant terms from a three-phase instantaneous currents is presented in the following subsection.

### 2.2 SoCR implementation

The calculation of the symmetrical components is usually presented using the operator *a*. This operator can be instantaneously calculated using Equation (3) [32], [33].

(3) $$a=e^{j\frac{2\pi }{3}}=e^{j(\frac{\pi }{2}+\frac{\pi }{6})}=-\frac{1}{2}+\frac{\sqrt{3}}{2}e^{j\frac{\pi }{2}}$$

Instantaneous symmetrical components decomposition is done by means of Equations (4), (5), and (6), as it was presented in [32], where the signal with the super-index $\frac{\pi }{2}$ is related to the signal with $\frac{\pi }{2}$ phase-lag.

(4) $$I_{1}=\left[\begin{array}{c} i_{1_{a}}\\ i_{1_{b}}\\ i_{1_{c}} \end{array}\right]=\frac{1}{3}\left[\begin{array}{ccc} 1 & -0.5 & -0.5\\ -0.5 & 1 & -0.5\\ -0.5 & -0.5 & 1 \end{array}\right]\left[\begin{array}{c} i_{a}\\ i_{b}\\ i_{c} \end{array}\right]-\frac{\sqrt{3}}{6}\left[\begin{array}{ccc} 0 & -1 & 1\\ 1 & 0 & -1\\ -1 & 1 & 0 \end{array}\right]\left[\begin{array}{c} i_{a}^{\frac{\pi }{2}}\\ i_{b}^{\frac{\pi }{2}}\\ i_{c}^{\frac{\pi }{2}} \end{array}\right]$$

(5) $$I_{2}=\left[\begin{array}{c} i_{2_{a}}\\ i_{2_{b}}\\ i_{2_{c}} \end{array}\right]=\frac{1}{3}\left[\begin{array}{ccc} 1 & -0.5 & -0.5\\ -0.5 & 1 & -0.5\\ -0.5 & -0.5 & 1 \end{array}\right]\left[\begin{array}{c} i_{a}\\ i_{b}\\ i_{c} \end{array}\right]+\frac{\sqrt{3}}{6}\left[\begin{array}{ccc} 0 & -1 & 1\\ 1 & 0 & -1\\ -1 & 1 & 0 \end{array}\right]\left[\begin{array}{c} i_{a}^{\frac{\pi }{2}}\\ i_{b}^{\frac{\pi }{2}}\\ i_{c}^{\frac{\pi }{2}} \end{array}\right]$$

(6) $$I_{0}=\left[\begin{array}{c} i_{0_{a}}\\ i_{0_{b}}\\ i_{0_{c}} \end{array}\right]=\left[\begin{array}{c} i_{0}\\ i_{0}\\ i_{0} \end{array}\right]=\frac{1}{3}\left[\begin{array}{c} i_{a}+i_{b}+i_{c}\\ i_{a}+i_{b}+i_{c}\\ i_{a}+i_{b}+i_{c} \end{array}\right]$$

The generation of the modified zero-sequence system, $I_{0m}$, is showed in Equation (7), in which a balanced system of positive-sequence is obtained adding a phase delay of $\frac{2\pi }{3}$ to $i_{0_{b}}$, and a phase delay of $\frac{4\pi }{3}$ to $i_{0_{c}}$. The approach was presented in [30], [29], [34], being based in the method developed in [32], [33]. It is important to highlight that $i_{0_{a}}=i_{0m_{a}}=i_{0}$ because when applying the *dq* transformation in Equation (7), *d* and *q* components corresponding to $i_{0m_{a}}$ are equal to $i_{0_{a}}$ and this particularly phenomenon allows finding the currents peak values.

(7) $$I_{0m}=\left[\begin{array}{c} i_{0m_{a}}\\ i_{0m_{b}}\\ i_{0m_{c}} \end{array}\right]=\left[\begin{array}{c} i_{0}\\ \frac{1}{2}(i_{0})-\frac{\sqrt{3}}{2}\left(i_{0}^{\frac{\pi }{2}}\right)\\ \frac{1}{2}(i_{0})+\frac{\sqrt{3}}{2}\left(i_{0}^{\frac{\pi }{2}}\right) \end{array}\right]$$

The fourth step shown in Fig. 2 consists of applying the positive *dq* transformation showed in Equation (8) to Equations (4) and (7) and the negative *dq* transformation showed in Equation (9) to Equation (5) [35].

(8) $$T_{abc\to dq}^{1}=\frac{2}{3}\left[\begin{array}{ccc} \cos\left(\omega t\right) & \cos\left(\omega t-\frac{2\pi }{3}\right) & \cos\left(\omega t-\frac{4\pi }{3}\right)\\ -\sin\left(\omega t\right) & -\sin\left(\omega t-\frac{2\pi }{3}\right) & -\sin\left(\omega t-\frac{4\pi }{3}\right) \end{array}\right]$$

(9) $$T_{abc\to dq}^{2}=\frac{2}{3}\left[\begin{array}{ccc} \cos\left(\omega t\right) & \cos\left(\omega t+\frac{2\pi }{3}\right) & \cos\left(\omega t+\frac{4\pi }{3}\right)\\ -\sin\left(\omega t\right) & -\sin\left(\omega t+\frac{2\pi }{3}\right) & -\sin\left(\omega t+\frac{4\pi }{3}\right) \end{array}\right]$$

Once the procedure for obtaining the SoCR has been presented, Equations for the six CR calculation are presented in Equation (10).

(10) $$\left[\begin{array}{c} i_{1_{d}}\\ i_{1_{q}} \end{array}\right]=T_{abc\to dq}^{1}\left[\begin{array}{c} i_{1_{a}}\\ i_{1_{b}}\\ i_{1_{c}} \end{array}\right];\left[\begin{array}{c} i_{2_{d}}i_{2_{q}} \end{array}\right]=T_{abc\to dq}^{2}\left[\begin{array}{c} i_{2_{a}}\\ i_{2_{b}}\\ i_{2_{c}} \end{array}\right];\left[\begin{array}{c} i_{0m_{d}}\\ i_{0m_{q}} \end{array}\right]=T_{abc\to dq}^{1}\left[\begin{array}{c} i_{0m_{a}}\\ i_{0m_{b}}\\ i_{0m_{c}} \end{array}\right]$$

The *dq* transformation of a balanced three-phase system, as it is the case of $I_{1}$, $I_{2}$, and $I_{0m}$ return constant values. Due to this, each symmetrical component of Equation (10) can be represented in complex values as shown in Equation (11), using the scalar $\left(-\frac{1}{2}+j\frac{\sqrt{3}}{2}\right)$ for generating $\frac{2\pi }{3}$ of phase-lag, and $\left(-\frac{1}{2}-j\frac{\sqrt{3}}{2}\right)$ for generating $\frac{4\pi }{3}$ of phase-lag.

(11) $$I_{1}=\left[\begin{array}{c} i_{1_{d}}+ji_{1_{q}}\\ \left(-\frac{1}{2}-j\frac{\sqrt{3}}{2})(i_{1_{d}}+ji_{1_{q}}\right)\\ \left(-\frac{1}{2}+j\frac{\sqrt{3}}{2})(i_{1_{d}}+ji_{1_{q}}\right) \end{array}\right];I_{2}=\left[\begin{array}{c} i_{2_{d}}+ji_{2_{q}}\\ (-\frac{1}{2}+j\frac{\sqrt{3}}{2})(i_{2_{d}}+ji_{2_{q}}\\ \left(-\frac{1}{2}-j\frac{\sqrt{3}}{2})(i_{2_{d}}+ji_{2_{q}}\right) \end{array}\right];I_{0}=\left[\begin{array}{c} i_{0_{d}}+ji_{0_{q}}\\ i_{0_{d}}+ji_{0_{q}}\\ i_{0_{d}}+ji_{0_{q}} \end{array}\right]$$

Given the SoCR procedure, the six constant references can be concatenated as the vector shown in Equation (12), that facilitates its mathematical manipulation when writing the optimization problem for a simulator or a microcontroller. The term *dev* represents the electrical output line of a device where SoCR is applied, where the types of devices can be classified attending to the power flows as follows: generator (PV or wind), consumption of energy (loads), and bidirectional devices like the BESS or the same MG.

(12) $${\overset{\to }{CR}}^{dev}={\left[\begin{array}{c} i_{1_{d}}\\ i_{1_{q}}\\ i_{2_{d}}\\ i_{2_{q}}\\ i_{0m_{d}}\\ i_{0m_{q}} \end{array}\right]}_{dev}$$

Current term denoted as $i_{1_{d}}$ is related to the fundamental positive-sequence active power, so the rest of terms defined in Equation (13) represent the non-efficient current terms in the device and can be used as compensating current vector if are supplied by EGs to reduce in the MG the adverse effects produced by the flows of the non-efficient load current terms.

(13) $${{\overset{\to }{CR}}^{dev}}_{N-eff}={\left[\begin{array}{c} i_{1_{q}}\\ i_{2_{d}}\\ i_{2_{q}}\\ i_{0m_{d}}\\ i_{0m_{q}} \end{array}\right]}_{dev}$$

### 2.3 Peak currents calculation

Compensation of fundamental non-efficient load current terms using distributed EGs must be done considering the rated maximum current of the EGs and without affecting its capacity as a source of energy in the MG. The peak value of the compensating currents assigned to the EGs must be controlled to avoid overcurrents. Each peak current is calculated using Equation (14), where $I_{1}$, $I_{2}$ and $I_{0}$ are written as function of $\overset{\to }{CR}$ terms, as it is presented in Equation (11).

(14) $$I=I_{1}+I_{2}+I_{0}$$

Equation (15) permits to calculate the maximum values for $i_{a}$, $i_{b}$, and $i_{c}$ as a function of direct and quadrature components. This expression is useful for representing linear unbalanced systems in terms of the six $\overset{\to }{CR}$ proposed in this paper.

(15) $$\left[\begin{array}{c} I_{a_{dq}}\\ I_{b_{dq}}\\ I_{c_{dq}} \end{array}\right]=\left[\begin{array}{c} i_{1_{d}}+ji_{1_{q}}+i_{2_{d}}+ji_{2_{q}}+i_{0_{d}}+ji_{0_{q}}\\ \left(-\frac{1}{2}-j\frac{\sqrt{3}}{2}\right)(i_{1_{d}}+ji_{1_{q}})+\left(-\frac{1}{2}+j\frac{\sqrt{3}}{2}\right)(i_{2_{d}}+ji_{2_{q}})+i_{0_{d}}+ji_{0_{q}}\\ \left(-\frac{1}{2}+j\frac{\sqrt{3}}{2}\right)(i_{1_{d}}+ji_{1_{q}})+\left(-\frac{1}{2}-j\frac{\sqrt{3}}{2}\right)(i_{2_{d}}+ji_{2_{q}})+i_{0_{d}}+ji_{0_{q}} \end{array}\right]$$

Square peak values are calculated using Equation (16). Square values are used to avoid the use of square roots in the optimization problem which yields to a non-convex problem.

(16) $$\left[\begin{array}{c} {\left(I_{a}\right)}^{2}\\ {\left(I_{b}\right)}^{2}\\ {\left(I_{c}\right)}^{2} \end{array}\right]=\left[\begin{array}{c} {\left(i_{1_{d}}+i_{2_{d}}+i_{0_{d}}\right)}^{2}+{\left(i_{1_{q}}+i_{2_{q}}+i_{0_{q}}\right)}^{2}\\ {\left(-\frac{1}{2}i_{1_{d}}-\frac{\sqrt{3}}{2}i_{1_{q}}-\frac{1}{2}i_{2_{d}}+\frac{\sqrt{3}}{2}i_{2_{q}}+i_{0_{d}}\right)}^{2}+{\left(-\frac{1}{2}i_{1_{q}}+\frac{\sqrt{3}}{2}i_{1_{d}}-\frac{1}{2}i_{2_{q}}-\frac{\sqrt{3}}{2}i_{2_{d}}+i_{0_{q}}\right)}^{2}\\ {\left(-\frac{1}{2}i_{1_{d}}+\frac{\sqrt{3}}{2}i_{1_{q}}-\frac{1}{2}i_{2_{d}}-\frac{\sqrt{3}}{2}i_{2_{q}}+i_{0_{d}}\right)}^{2}+{\left(-\frac{1}{2}i_{1_{q}}-\frac{\sqrt{3}}{2}i_{1_{d}}-\frac{1}{2}i_{2_{q}}+\frac{\sqrt{3}}{2}i_{2_{d}}+i_{0_{q}}\right)}^{2} \end{array}\right]$$

## 3 Optimization problem formulation for non-efficient load current distribution between EGs in a grid-tied MG

**Figure 3:**
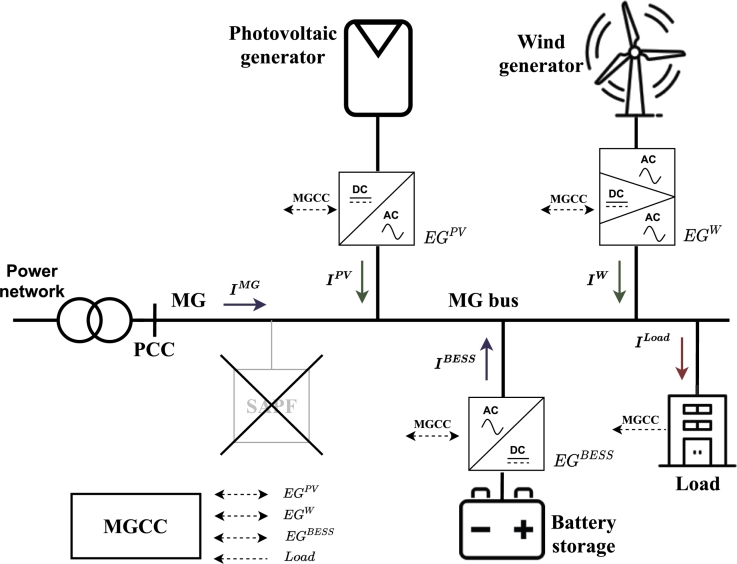
MG topology used for optimal non-efficient load current term distribution among EGs.

It is important to present how SoCR is used for distribution of non-efficient fundamental current among EGs in an MG. In the following sections the grid-tied MG depicted in Fig. 3 is considered. This MG is composed of loads (a residential building), a PV system, a wind system, and a BESS. The MG operation is managed by means of the MGCC. The PV system, the wind system, and the BESS are the so-called Energy Gateways (EGs), operating as energy interfaces between the non-conventional sources and the MG. The main function of these EGs is, in addition to supply fundamental positive-sequence active power into the MG, to compensate all the fundamental non-efficient load current terms. The problem that arises is how to establish the control set-points of each EG to distribute the compensation of the non-efficient currents flowing through the MG. The SAPF shown as removed in Fig. 3 represents that the MG does not need the SAPF to compensate the fundamental non-efficient load current terms, a role that will be taken over by the existing EGs in the MG.

In general, considering an MG connected to a power network with which it exchanges currents represented by ${\overset{\to }{CR}}^{MG}$, with *n* EGs (*nEG*) and *m* loads (mLoad), from the SoCR perspective, fulfills the relationship of Equation (17). It is important to remark that Equation (17) assumes that current flows from the power network to the MG, from the EGs to the MG, and from the MG to the load, as it is presented in Fig. 3. This assumption states the sign of the terms appearing in the following equation.

(17) $$\sum_{m=1}^{mLoad}{\overset{\to }{CR}}^{mLoad}=\sum_{n=1}^{nEG}{\overset{\to }{CR}}^{nEG}+{\overset{\to }{CR}}^{MG}$$

Where ${\overset{\to }{CR}}^{MG}$, ${\overset{\to }{CR}}^{nEG}$, and ${\overset{\to }{CR}}^{mLoad}$ are respectively representing the current terms in the MG, the EGs, and the loads. Equation (17) corresponds to current Kirchhoff's law.

With regards to ${i_{1_{d}}}^{MG}$, a positive value means that the power network is providing fundamental positive-sequence active power to the MG, a negative value means that the power network is receiving fundamental positive-sequence active power, and a zero value means there is not energy exchange between MG and power network; i.e., the MG is self-sufficient. $\overset{\to }{CR}$ calculation considers the direction of the current flow, therefore, it is important to consider sensor polarity and EG connection. As ${\overset{\to }{CR}}_{N-eff}$ are related to fundamental non-efficient current terms, the MGCC operates to reduce or cancel the terms contained in ${{\overset{\to }{CR}}^{MG}}_{N-eff}$ in the proximity of the loads that demand ${{\overset{\to }{CR}}^{Load}}_{N-eff}$. To achieve this objective, ${{\overset{\to }{CR}}^{nLoad}}_{N-eff}$ terms must be distributed among the EGs present in the MG trying to reach an operating condition in which ${{\overset{\to }{CR}}^{MG}}_{N-eff}$ are equal to zero, existing only $i_{1_{d}}$ upstream the MG.

Regarding the non-efficient load currents in an MG, the most common solution to improve power quality in an electrical power system is the inclusion of an SAPF in the installation, in the surroundings of the loads that demand the non-efficient current terms. The approach developed in this work uses the EGs present in the MG to achieve the same compensation characteristics as the SAPF-based solution. The distribution of the non-efficient load current terms between the EGs is achieved by considering the remaining current capacities of the EGs after supplying the available fundamental positive-sequence active power, which could cover the active power demanded by the loads or could be exported to other parts of the power network. This approach has two main advantages: 1) it avoids the installation of SAPFs, reducing costs and simplifying the complexity of the MG, and 2) it takes advantage of the flexibility of the EGs to improve the power quality of the MG, even in the case of partial compensation of the non-efficient load currents.

The distribution of ${{\overset{\to }{CR}}^{Load}}_{N-eff}$ between the EGs connected to the MG requires to consider the following: 1) the EGs assigned to the compensation must have some remaining current capacity, and 2) the maximum current capacity of the EGs may not be exceeded in any case, otherwise, some of the devices of the MG may be damaged and the MG could be disconnected. The $\overset{\to }{CR}$ are obtained by means of the *dq* transformation and symmetrical components decomposition, which are linear transformations. Therefore, the SoCR satisfies the superposition property of linear systems and Equation (17) can be applied for the distributed compensation of the non-efficient load current terms because the inverse operation of each transformation is guaranteed.

The optimization problem starts considering the current balance established by Equation (17). Because $i_{1_{d}}$ term is related to the fundamental positive-sequence active power, it does not matter whether it is supplied by the power network, the EGs, or as combination of both. It is normally preferred that the EGs supply this $i_{1_{d}}$ to maximize the utilization of the MG, and it is common for the EGs to have remaining current capacity to also supply ${\overset{\to }{CR}}_{N-eff}$ (in whole or in part) to the MG. In order to enhance the power quality in the MG, ${{\overset{\to }{CR}}^{MG}}_{N-eff}$ must be equal or as close as possible to $\overset{\to }{0}$. Excluding $i_{1_{d}}$ in equation (17), the following equation arises:

(18) $${{\overset{\to }{CR}}^{MG}}_{N-eff}=\sum_{n=1}^{nEG}{{\overset{\to }{CR}}^{nEG}}_{N-eff}-\sum_{m=1}^{mLoad}{{\overset{\to }{CR}}^{mLoad}}_{N-eff}=\overset{\to }{0}$$

where ${{\overset{\to }{CR}}^{mLoad}}_{N-eff}$ are the known values (they can be either measured or estimated) and ${{\overset{\to }{CR}}^{nEG}}_{N-eff}$ are the variables to be chosen to satisfy equality. Equation (18) can be understood as a distance measure between ${{\overset{\to }{CR}}^{nEG}}_{N-eff}$ and ${{\overset{\to }{CR}}^{mLoad}}_{N-eff}$ and could be directly used as the objective function in an optimization problem where the goal would be to find ${{\overset{\to }{CR}}^{nEG}}_{N-eff}$ values such that ${{\overset{\to }{CR}}^{MG}}_{N-eff}$ approach to $\overset{\to }{0}$. However, quadratic functions are commonly preferred as objective functions in optimization problems because their potential to result in convex optimization problems. In this sense, the squared Euclidean Distance, denoted as ${\left\|\cdot \right\|}^{2}$, is applied to the above equality, looking for a parametric equation that can be turned into a constrained optimization problem, as shown in Equation (19).

(19) $$J\left(\sum_{n=1}^{nEG}{{\overset{\to }{CR}}^{nEG}}_{N-eff}\right)={\left\|\sum_{n=1}^{nEG}{{\overset{\to }{CR}}^{nEG}}_{N-eff}-\sum_{m=1}^{mLoad}{{\overset{\to }{CR}}^{mLoad}}_{N-eff}\right\|}^{2}$$

To complete the optimization problem formulation, some additional constraints must be defined. These constraints account for the peak current being delivered by the EGs, that have to be smaller than its maximum current capacity ($I_{lim}^{nEG}$). Consequently, the constrained optimization problem to be solved can be written by means of the objective function presented in Equation (20) and their respective constrains for EGs, defined in Equation (21).

(20) $$\begin{array}{cccc} & \underset{\sum_{n=1}^{nEG}{{\overset{\to }{CR}}^{nEG}}_{N-eff}}{\text{argmin}} & & {\left\|\sum_{n=1}^{nEG}{{\overset{\to }{CR}}^{nEG}}_{N-eff}-\sum_{m=1}^{mLoad}{{\overset{\to }{CR}}^{mLoad}}_{N-eff}\right\|}^{2} \end{array}$$

(21) $$\begin{array}{cccc} & & & {\left[\begin{array}{c} {\left(I_{a}\right)}^{2}\\ {\left(I_{b}\right)}^{2}\\ {\left(I_{c}\right)}^{2} \end{array}\right]}_{nEG}<{\left(I_{lim}^{nEG}\right)}^{2}\left[\begin{array}{c} 1\\ 1\\ 1 \end{array}\right] \end{array}$$

**Figure 4:**
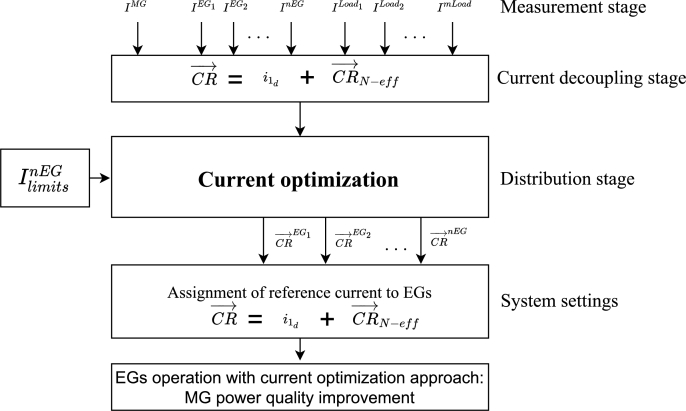
Flowchart for the distribution of non-efficient current terms using the proposed optimization procedure.

Fig. 4 presents the flowchart of the proposed procedure for ${{\overset{\to }{CR}}^{Load}}_{N-eff}$ distribution. The first step consists of measuring the currents in the EGs, the load, and the MG. After that, the current decoupling by means of SoCR is applied and ${{\overset{\to }{CR}}^{Load}}_{N-eff}$ are obtained. It is necessary to include the $I^{MG}$ current for validating if the system works inefficiently. The distribution of ${{\overset{\to }{CR}}^{Load}}_{N-eff}$ is performed in the MGCC considering the $I_{limits}^{nEG}$ and the optimal distribution proposed in Equation (20). The second step is an optimization current block that uses the argmin function (please see, Equation (20)). In the optimization process, the first argmin value corresponding to the ${{\overset{\to }{CR}}^{EGs}}_{N-eff}$ measured in the EGs is used for beginning the iterations. In the third step, $\overset{\to }{CR}$ are respectively assigned to the EGs, which change their set-points to improve the power quality in the MG. Fourth block consists on converting the obtained $\overset{\to }{CR}$ in the *abc* reference frame applying the inverse process of figure 2. For doing this, first it is applied positive sequence inverse *dq* transformation to [$i_{0m_{d}}$, $i_{0m_{q}}$], [$i_{1_{d}}$, $i_{1_{q}}$] and [$i_{2_{d}}$, $i_{2_{q}}$]. Second, it is obtained the zero-sequence components ($i_{0_{a}}=i_{0_{ma}}$, $i_{0_{b}}=i_{0_{ma}}$, and $i_{0_{c}}=i_{0_{ma}}$). Third, it adds the symmetrical components to find the instantaneous value that each inverter must have. Fourth, it establishes the instantaneous references to each EG. The first and third blocks must have the same expression because the first one decomposes each signal measured from the EGs, the loads, and the MG, while the third block reconstructs the signal that must follow each EG. It can be noted that the inputs and outputs of each block are different. The current of the MG is measured to monitor, and the current of each EG is measured to determine the active power and use the last distribution of inefficiencies in the optimization algorithm that requires a starting point.

## 4 Results

The methodology for distributing ${{\overset{\to }{CR}}^{Load}}_{N-eff}$ between EGs is validated in a grid-tied MG with a nominal voltage equal to 120 V (line to neutral) at 60 Hz, only including the fundamental positive-sequence voltage components. The target peak value for the instantaneous output current of PV and BESS is 35 A, equivalent to 8909 VA, while for wind generation it is set at 25 A, equivalent to 6364 VA. This is a common scenario for medium power size MGs and takes relevance when linear unbalanced loads overload one or two phases. Fig. 3 depicts the scheme of the MG considered to perform the simulations in OpenModelica platform. The load configuration and the fundamental positive-sequence active power supplied by the EGs are kept constant along the different simulations. The energy flow between the MG and the power network shown in Fig. 3 only contains fundamental positive-sequence power, although attending to the EGs conditions, the sign of this power can vary, representing that the MG operates demanding or supplying energy to the power network. The linear unbalanced load used in the simulations is composed of three resistive-inductive loads in series connected between phase and neutral with the following values: $R_{a}$*=1.57* Ω, $L_{a}$*=3.5 mH*; $R_{b}$*=3.2* Ω, $L_{b}$ *=1.188 mH*; and $R_{c}$*=1.439* Ω, $L_{c}$ *=1.925 mH*. The connection of the load is done at *t*=0.05 s for all the simulated scenarios. Table 1 corresponds to the current values after MG load connection (*t*> 0.05 s) that includes the peak values and their corresponding decomposition in the six constant references.

**Table 1:** Peak values for ***I***^*Load*^ and ${\overset{\to }{CR}}^{Load}$ after the short transient of the load connection (*t*> 0.05 s).

	Current term	Peak value (A)
***I***^*Load*^	${I_{a}}^{Load}$	81.85
	${I_{b}}^{Load}$	52.52
	${I_{c}}^{Load}$	105.24

${\overset{\to }{CR}}^{Load}$	$i_{1_{d}}^{Load}$	69.42
	$i_{1_{q}}^{Load}$	-35.89
	$i_{2_{d}}^{Load}$	-15.07
	$i_{2_{q}}^{Load}$	-20.73
	$i_{0m_{d}}^{Load}$	8.11
	$i_{0m_{q}}^{Load}$	3.58

**Figure 5:**
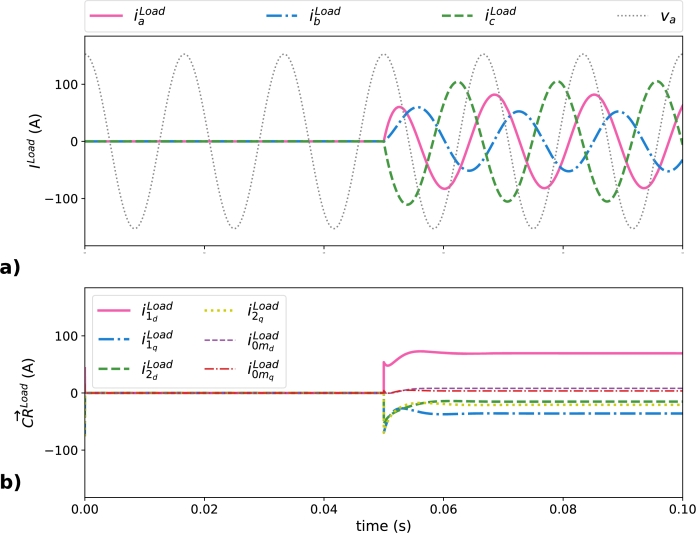
Current demanded by the load in all scenarios: a) instantaneous current terms represented by ***I***^*Load*^, and b) ${\overset{\to }{CR}}^{Load}$ values obtained with the SoCR procedure.

Fig. 5 depicts MG load current and their corresponding decomposition in the six constant references (connection of load occurs in *t*> 0.05 s). Fig. 5a shows the instantaneous load currents that are represented by $I^{Load}$ ($i_{a}^{Load}$, ${i_{b}}^{Load}$*, and* $i_{c}^{Load}$) and Fig. 5b shows ${\overset{\to }{CR}}^{Load}$ ($i_{1_{d}}$, $i_{1_{q}}$, $i_{2_{d}}$, $i_{2_{d}}$, $i_{0_{md}}$, and $i_{0_{mq}}$). A proportional signal of $v_{a}$ is added in the following time domain plots to include the origin of angles of the three-phase system. After the load connection and the short transient due to the inductive parts of the load, $I^{Load}$ terms reach their steady-state values which yields to constant values for the ${\overset{\to }{CR}}^{Load}$ obtained with the SoCR decomposition, validating the SoCR suitability to represent linear unbalanced systems. It is observed that the term related to the active power ($i_{1_{d}}$) is the largest of all the $\overset{\to }{CR}$ obtained and the load unbalance is clearly identified since all the ${\overset{\to }{CR}}_{N-eff}$ terms are non-zero.

In the baseline scenario one EG supplies all ${{\overset{\to }{CR}}^{Load}}_{N-eff}$ terms to improve the power quality in the MG, being presented to validate the suitability of the proposed SoCR. Later, other scenarios with different ${{\overset{\to }{CR}}^{Load}}_{N-eff}$ distributions are proposed, where the last of the scenarios corresponds to the optimal distribution of ${{\overset{\to }{CR}}^{Load}}_{N-eff}$ terms between EGs obtained by means of Equation (20). In the performed simulations it is analyzed how the limits established for each EG are exceeded, and how the distribution of non-efficient load current terms are improved. The scenarios considered in our study are summarized as follows:

1. Baseline scenario: the MG is working with only BESS and load, so ${{\overset{\to }{CR}}^{Load}}_{N-eff}$ are assigned to the BESS controller.
2. Balanced distribution: ${{\overset{\to }{CR}}^{Load}}_{N-eff}$ are shared equally between EGs, so ${{\overset{\to }{CR}}^{PV}}_{N-eff}=$ ${{\overset{\to }{CR}}^{W}}_{N-eff}=$ ${{\overset{\to }{CR}}^{BESS}}_{N-eff}=$ $\frac{1}{3}{{\overset{\to }{CR}}^{Load}}_{N-eff}$.
3. Proportional distribution: the MGCC control algorithm considers the remaining capacity of each EG and distributes ${{\overset{\to }{CR}}^{Load}}_{N-eff}$ proportionally between the EGs.
4. Optimal distribution: ${{\overset{\to }{CR}}^{Load}}_{N-eff}$ are distributed between EGs using the optimization model proposed in Equation (20).

Table 2 shows the steady-state peak values of $i_{1_{d}}$ in the four considered scenarios. The first column corresponds to the EGs of the system (Load, PV, W, BESS, and MG). Second and third columns correspond to $i_{1_{d}}$ for baseline scenario before and after load connection in t=0.05 s respectively. Fourth and fifth columns correspond to $i_{1_{d}}$ for the other scenarios before and after load connection in t=0.05 s respectively.

**Table 2:** Steady-state peak values of $i_{1_{d}}$ in the four considered scenarios.

Evaluated device	Scenarios			
	1. Baseline scenario		**Table:** 2. Balanced distribution 3. Proportional distribution 4. Optimal distribution	
	*t*<0.05 s	*t*>0.05 s	*t*<0.05 s	*t*>0.05 s
${i_{1_{d}}}^{Load}$ (A)	0	69,42	0	69,42
${i_{1_{d}}}^{PV}$ (A)	0	0	30.00	30.00
${i_{1_{d}}}^{W}$ (A)	0	0	17.00	17.00
${i_{1_{d}}}^{BESS}$ (A)	35.00	35.00	-12.00	-12.00
${i_{1_{d}}}^{MG}$ (A)	**-35.00**	**34,42**	**-35.00**	**34,42**

In the baseline scenario, before the load connection, the BEES supply to the MG only fundamental positive-sequence active current terms. After the load connection, both BESS and MG supply the current terms demanded by the load, being the responsibility of the BESS to deliver the non-efficient components demanded by the load. All the scenarios simulated are considering the same values of $i_{1_{d}}$ in the MG, before and after the load connection, as it is shown in the last row in Table 2. The summation of $i_{1_{d}}$ of EGs in all the scenarios is equal to ${i_{1_{d}}}^{BESS}$ in the baseline scenario, as shown by the values of the last two columns in Table 2, before and after load connection respectively. The ${i_{1_{d}}}^{PV}$=30 A was established near the $I_{Limit}^{PV}$ looking for a critical distribution problem of ${{\overset{\to }{CR}}^{Load}}_{N-eff}$ values. Wind power was decreased in respect of PV and BESS looking for a hard restriction in the distribution of ${{\overset{\to }{CR}}^{Load}}_{N-eff}$ between EGs and to represent a real MG with different EGs capacities. The values of ${i_{1_{d}}}^{W}$=17 A and ${i_{1_{d}}}^{BESS}=$-12 A are set, with the condition that the total sum of the EGs' currents is 35 A and with the BESS absorbing power for the MG.

**Figure 6:**
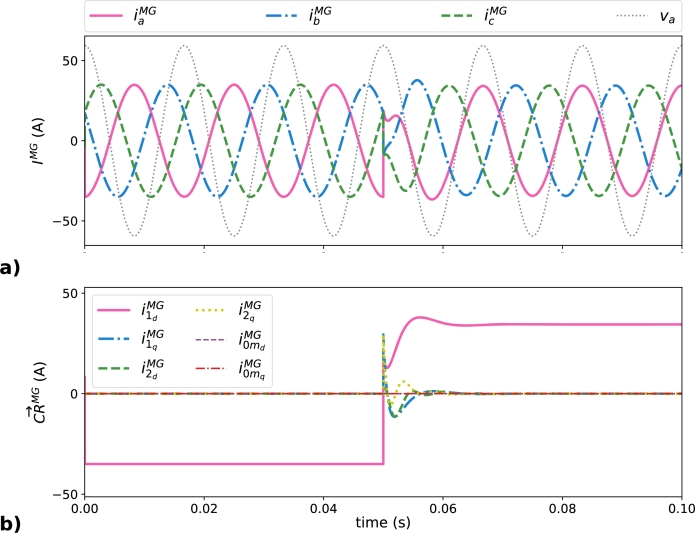
Current terms in the MG in all scenarios: a) instantaneous current terms represented by ***I***^*MG*^, and b) ${\overset{\to }{CR}}^{MG}$ values obtained with the SoCR procedure.

$I^{MG}$ and ${\overset{\to }{CR}}^{MG}$ are presented in Fig. 6. All the peak values in Fig. 6 are equal to ${i_{1_{d}}}^{MG}$ shown in Table 2. During the interval in which the load is disconnected (*t*<0.05 s), ${i_{1_{d}}}^{BESS}$ is supplying current to the MG, that operates as a load that injects the active power supplied by the BESS into the power network, as it is shown by the negative sign of ${i_{1_{d}}}^{MG}=$ -35 A in Table 2, the 180º phase shift between $v_{a}$ and $i_{a_{MG}}$ in Fig. 6a, or the negative value of ${i_{1_{d}}}^{MG}$ in Fig. 6b. After the load connection (*t*>0.05 s), the MG supply ${i_{1_{d}}}^{MG}$=34.42 A, that summed up with $i_{1_{d}}$ from the BESS (a total of 35 A) permits covering the load current ${i_{1_{d}}}^{Load}$ =69.42 A. The null phase shift between $v_{a}$ and $i_{a}^{MG}$ in Fig. 6a or the positive value of ${i_{1_{d}}}^{MG}$ in Fig. 6b clearly shows that the power network is operating as a source of energy for *t*>0.05 s. In any case, the MG is operating with the best power quality, without non-efficient current terms flowing through it, as it is shown in Fig. 6b (all ${{\overset{\to }{CR}}^{MG}}_{N-eff}$ are null).

### 4.1 Baseline scenario

This baseline scenario demonstrates that the SoCR method is a suitable tool for the decomposing of fundamental unbalanced currents to implement selective compensation strategies of the fundamental non-efficient load current terms represented in ${{\overset{\to }{CR}}^{Load}}_{N-eff}$, taking advantage of the flexibility of EGs. It is assumed that only the BESS is available in the MG and has the capability to simultaneously supply $i_{1_{d}}=35$ A to the MG and to compensate all ${{\overset{\to }{CR}}^{Load}}_{N-eff}$.

From the measurement of the currents in the load and their subsequent decomposition following Equations (4) to (10), the MGCC sets ${{\overset{\to }{CR}}^{load}}_{N-eff}$ terms in the BESS controller. Table 3 contains the six reference values ($\overset{\to }{CR}$) after the load connection in *t*=0.05 s in the baseline scenario for load (second column), BESS (third column), and MG (fourth column). Table 3 shows how ${{\overset{\to }{CR}}^{BEES}}_{N-eff}$ are equal to ${{\overset{\to }{CR}}^{Load}}_{N-eff}$, fulfilling Equation (18). $i_{1_{d}}^{Load}$ is supplied between the BESS ($i_{1_{d}}^{BESS}=35$ A), keeping the value scheduled by the MGCC, and the MG ($i_{1_{d}}^{MG}=34.42$ A). As can be seen in the last column of Table 3, the power network is only supplying $i_{1_{d}}$, with a null value in all the non-efficient current terms, as corresponds to the verification of Equation (17) and as shown in Fig. 6.

**Table 3:** $\overset{\to }{CR}$ after load connection (*t*>0.05 s) in the baseline scenario.

$\overset{\to }{CR}$	${\overset{\to }{CR}}^{Load}$	${\overset{\to }{CR}}^{BESS}$	${\overset{\to }{CR}}^{MG}$
$i_{1_{d}}$ (A)	69.4	35	34.42
$i_{1_{q}}$ (A)	-35.89	-35.89	0
$i_{2_{d}}$ (A)	-15.07	-15.07	0
$i_{2_{q}}$ (A)	-20.73	-20.73	0
$i_{0m_{d}}$ (A)	8.11	8.11	0
$i_{0m_{q}}$ (A)	3.58	3.58	0

**Figure 7:**
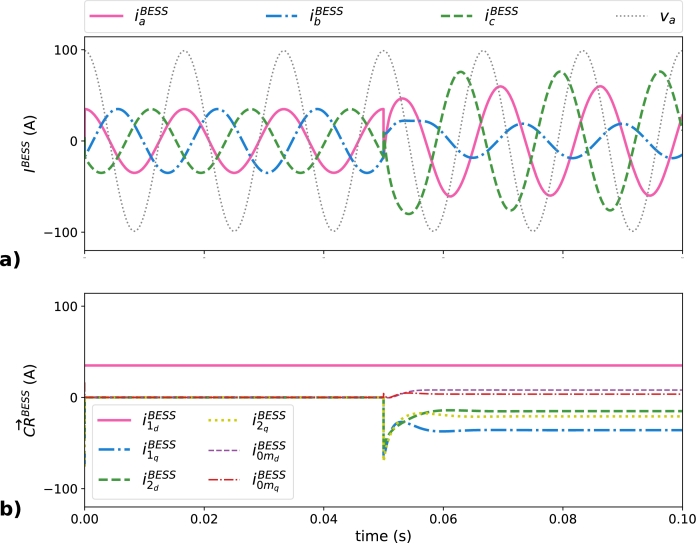
Current supplied by the BESS in the baseline scenario: a) ***I***^*BESS*^ and b) ${\overset{\to }{CR}}^{BESS}$.

Fig. 7 corresponds to the current supplied by the BESS and their corresponding decomposition in the six constant references for baseline scenario. Fig. 7a presents the BESS currents in the time domain. Until the load connection, the BESS is supplying a constant fundamental positive-sequence active power to the grid ($v_{a}$ and $i_{a}^{BESS}$ are in phase). After the load connection, the active power generated by the BESS remains at the same value, as can be observed by the value of $i_{1_{d}}^{BESS}$ which is constant and equal to 35 A during the whole simulation. The compensation of the non-efficient load current terms explains the presence of ${{\overset{\to }{CR}}^{BESS}}_{N-eff}$ after the load connection, as is showed in Fig. 7b. The BESS output currents in the time domain ($I^{BESS}$) are unbalanced and include phase shifts that compensates all ${{\overset{\to }{CR}}^{Load}}_{N-eff}$ terms. The main problem with these current waveforms is the maximum value of $i_{c}^{BESS}$, that arrives to 75.16 A (2.16 times greater than the target limiting current $I_{c}^{BESS}$=35 A), being this value the one that determines the apparent power rating of the EGs, although the BESS output currents in phases *a* or *b* are smaller. This means that if only one EG of the MG is supplying all ${{\overset{\to }{CR}}^{Load}}_{N-eff}$ at once, this device can be easily overloaded and consequently the operating condition leads to an EG outage and the MG would present a lower power quality.

Regarding the SoCR baseline simulation, the simulated results demonstrate how a three-phase system can be represented by means of constant references ($\overset{\to }{CR}$), with a perfect decoupling of the efficient term ($i_{1_{d}}$) from the non-efficient terms, represented by ${\overset{\to }{CR}}_{N-eff}$. The uncoupling between $i_{1_{d}}$ and ${\overset{\to }{CR}}_{N-eff}$ allows the distribution of the non-efficient current terms among the EGs present in the MG. The solution usually proposed in the literature consists of the inclusion of one shunt active power filter, as in [19], [22], although this solution requires an over-sized device that leads to over costs or a low use of the installed infrastructure capacity.

### 4.2 Balanced distribution scenario

**Figure 8:**
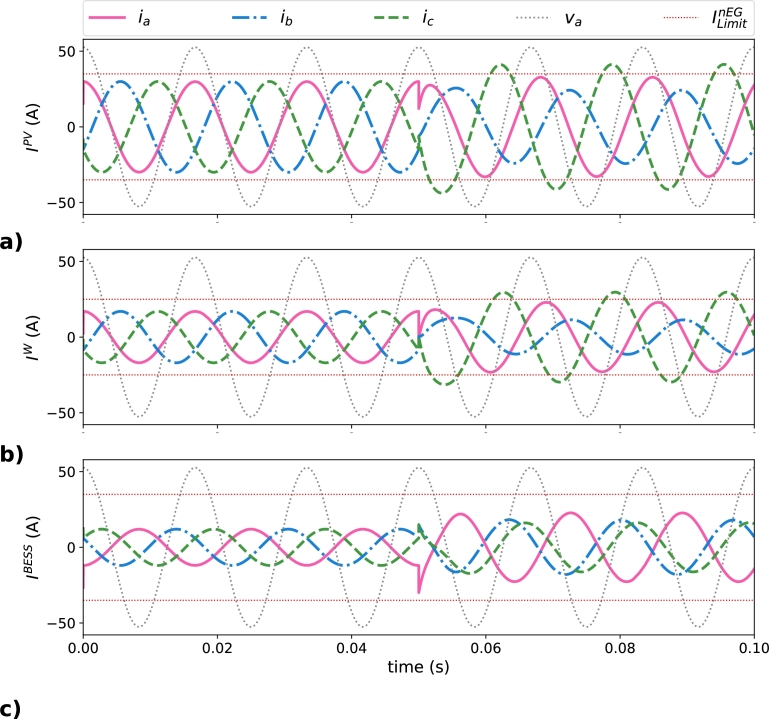
Current behavior of EGs in the balanced distribution scenario: a) ***I***^*PV*^, b) ***I***^*W*^, and c) ***I***^*BESS*^.

The balanced distribution scenario assumes that ${{\overset{\to }{CR}}^{Load}}_{N-eff}$, presented in Table 3, is equally distributed among the three EGs, verifying that ${{\overset{\to }{CR}}^{PV}}_{N-eff}=$ ${{\overset{\to }{CR}}^{W}}_{N-eff}=$ ${{\overset{\to }{CR}}^{BESS}}_{N-eff}=$ $\frac{1}{3}{{\overset{\to }{CR}}^{Load}}_{N-eff}$. The results obtained for this distribution approach are outlined in Table 4 and plotted in Fig. 8. The operating conditions detailed in Table 4 before the load connection, for t<0.05 s, are common to the following scenarios, matching with the values of $i_{1_{d}}$ shown in Table 2. Maximum peak current values of EGs during the simulation of this scenario are reported in Table 4 attending the load state. Negative values in Table 4 for BESS represent the storage of energy.

**Table 4:** Peak phase currents (in A) in the EGs in the balanced distribution scenario.

MG element	Phase current (A)	*t*<0.05 s	*t*>0.05 s
	${I_{a}}^{PV}$	30	32.33
***I***^*PV*^	${I_{b}}^{PV}$	30	24.29
(target=35 A)	${I_{c}}^{PV}$	30	**41.32**

	${I_{a}}^{W}$	17	22.95
***I***^*W*^	${I_{b}}^{W}$	17	11.42
(target=25 A)	${I_{c}}^{W}$	17	**29.63**

	${I_{a}}^{BESS}$	-12	-22.75
***I***^*BESS*^	${I_{b}}^{BESS}$	-12	-18.00
(target=35 A)	${I_{c}}^{BESS}$	-12	-16.15

Fig. 8 presents the currents of the EGs in the time domain. Fig. 8a corresponds to $I^{PV}$, Fig. 8b corresponds to $I^{W}$, and Fig. 8c corresponds to $I^{BESS}$; where $I_{Limit}^{nEG}$ is included in the plots as two horizontal dashed lines. Before the load connection $I^{PV}$ and $I^{W}$ are in phase with their corresponding phase voltage, as corresponds when EGs are generating only fundamental positive-sequence active power, while $I^{BESS}$ present a phase shift of 180º due to the BESS is storing energy. After the load connection and the distribution of ${{\overset{\to }{CR}}^{Load}}_{N-eff}$ between the EGs, the phase currents in the EGs are unbalanced and present different phase shifts. Furthermore, some phase currents of the EGs are exceeding their corresponding maximum rated current, denoted as $I_{Limit}^{nEG}$. The values that overcome the rated limits are $I_{c}^{PV}$, with 41.32 A for a $I_{Limit}^{PV}$ = 35 A, and $I_{c}^{W}$, with 29.63 A for a $I_{Limit}^{W}$ = 25 A, being both represented in bold characters in Table 4.

Regarding the balanced distribution scenario, results obtained demonstrate how ${{\overset{\to }{CR}}^{Load}}_{N-eff}$ distribution among EGs of an MG system can be done. The value of the maximum currents of all EGs in this scenario is smaller than with the obtained in the baseline scenario, where only one EG was compensating the non-efficient currents and its peak value of current in one phase was 75.6 A. Despite this, some instantaneous phase currents are exceeding the corresponding rated values set by $I_{Limit}^{nEG}$, so this distribution approach is not valid if the limits of operation of EGs are considered.

### 4.3 Proportional distribution scenario

**Figure 9:**
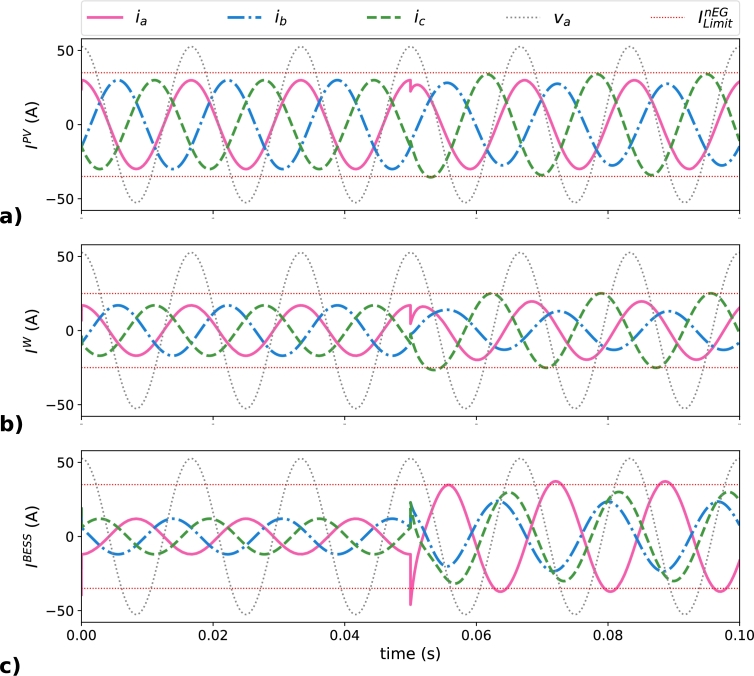
Instantaneous currents of EGs in the proportional distribution scenario 3: a) ***I***^*PV*^, b) ***I***^*W*^, and c) ***I***^*BESS*^.

To avoid the problem of exceeding the values of $I_{Limit}^{nEG}$, the proportional distribution approach contemplates the availability of each EGs in ${{\overset{\to }{CR}}^{Load}}_{N-eff}$ compensation. Considering the values shown in Table 5 before the load connection, the current availability for compensation is equal to 5 A, 8 A, and 23 A for PV, wind, and BESS respectively, resulting in compensation factors of 14% for PV (${{\overset{\to }{CR}}^{PV}}_{N-eff}=$ $0.14{{\overset{\to }{CR}}^{Load}}_{N-eff}$), 23% for wind (${{\overset{\to }{CR}}^{w}}_{N-eff}=$ $0.23{{\overset{\to }{CR}}^{Load}}_{N-eff}$), and 63% for BESS (${{\overset{\to }{CR}}^{BESS}}_{N-eff}=$ $0.63{{\overset{\to }{CR}}^{Load}}_{N-eff}$). Instantaneous currents of the EGs ($I^{PV}$, $I^{W}$, and $I^{BESS}$) are plotted in Fig. 9a, Fig. 9b, and Fig. 9c respectively; reporting in Table 5 the maximum values reached in their corresponding phases.

**Table 5:** Peak phase currents (in A) in the EGs in the proportional distribution scenario.

MG element	Phase current (A)	*t*<0.05 s	*t*>0.05 s
	${I_{a}}^{PV}$	30	27.55
***I***^*PV*^	${I_{b}}^{PV}$	30	29.95
(target=35 A)	${I_{c}}^{PV}$	30	34.08

	${I_{a}}^{W}$	17	**25.13**
***I***^*W*^	${I_{b}}^{W}$	17	13.05
(target=25 A)	${I_{c}}^{W}$	17	19.64

	${I_{a}}^{BESS}$	-12	**-37.59**
***I***^*BESS*^	${I_{b}}^{BESS}$	-12	-23.52
(target=35 A)	${I_{c}}^{BESS}$	-12	-30.09

As highlighted in Table 5 and can be seen in Fig. 9, after the load connection the PV generator is operating very close of $I_{Limit}^{PV}$ but $I_{a}^{BESS}=$ 37.59 A and $I_{a}^{W}=$ 25.13 A are exceeding their $I_{Limit}^{nEG}$ values, equal to 35 A and 25 A respectively. The results obtained show that with the proportional distribution scenario, the distribution of ${{\overset{\to }{CR}}^{Load}}_{N-eff}$ can establish peak current values not allowed for the EGs, which should be considered by the MGCC to avoid problems with the operation of the MG itself and to achieve the highest possible power quality with the available resources. Main advantage of the proportional distribution scenario approach with respect to the balanced distribution scenario is in the reduction of the over-currents in the EGs concerning to $I_{Limit}^{nEG}$, where $I_{a}^{BESS}=$ 37.59 A and $I_{a}^{W}=$ 25.13 A are replacing the values obtained with the previous scenario ($I_{c}^{PV}=$ 41.32 A and $I_{c}^{W}=$ 29.63 A). Despite this reduction in the peak currents, the proportional distribution scenario approach does not permit to operate the MG in its optimal conditions if the values of $I_{Limit}^{nEG}$ are considered, resulting in a ${{\overset{\to }{CR}}^{MG}}_{N-eff}$ not null, that is not the most efficient behavior of the MG.

### 4.4 Optimal distribution scenario

Previous approaches to distribute ${{\overset{\to }{CR}}^{Load}}_{N-eff}$ between EGs present problems if the values of $I_{Limit}^{nEG}$ are considered. The optimal distribution approach overcomes this situation by means of the optimization model presented in Equation (20). The proposed optimization method guarantees the best ${{\overset{\to }{CR}}^{Load}}_{N-eff}$ distribution among EGs without exceeding $I_{Limit}^{nEG}$. This optimization model requires setting a starting condition (SC), choosing to use the non-efficient current terms applied to the EGs after load connection in the proportional distribution scenario, as shown in Equation (22). This SC was selected because it is the nearest known value to the final solution. This optimization problem has three EGs, with five non-efficient current terms in each, so there are fifteen variables and fifteen constraints.

(22) $${{\overset{\to }{CR}}^{Load}}_{N-eff}={{\overset{\to }{CR}}^{PV}}_{N-eff}+{{\overset{\to }{CR}}^{W}}_{N-eff}+{{\overset{\to }{CR}}^{BESS}}_{N-eff}=(0.14+0.23+0.63)⁎{{\overset{\to }{CR}}^{Load}}_{N-eff}{\left[\begin{array}{c} -35.89\\ -15.07\\ -20.73\\ 8.11\\ 3.58 \end{array}\right]}_{Load}={\left[\begin{array}{c} -5.02\\ -2.10\\ -2.90\\ 1.13\\ 0.50 \end{array}\right]}_{PV_{SC}}+{\left[\begin{array}{c} -8.25\\ -3.46\\ -4.76\\ 1.86\\ 0.82 \end{array}\right]}_{W_{SC}}+{\left[\begin{array}{c} -22.61\\ -9.49\\ -13.05\\ 5.11\\ 2.25 \end{array}\right]}_{BESS_{SC}}$$

The optimization method proposed in Equation (20) was solved in Python, an open-source software, with Scipy library, which is based in Trust Region Methods presented in [36]. The optimal solution for non-efficient distribution, considering the selected SC detailed in Equation (22), is reported in Table 6, where the SoCR terms for each MG element after the load connection are reported as a SoCR vector ($\overset{\to }{CR}$). In this Table, the second column is the sum of the last four columns (${\overset{\to }{CR}}^{Load}$=${\overset{\to }{CR}}^{PV}$+${\overset{\to }{CR}}^{W}$+${\overset{\to }{CR}}^{BESS}$+${\overset{\to }{CR}}^{MG}$), fulfilling Equation (17).

**Table 6:** $\overset{\to }{CR}$ terms of optimal solution for *t*>0.05 s.

$\overset{\to }{CR}$	${\overset{\to }{CR}}^{Load}$	${\overset{\to }{CR}}^{PV}$	${\overset{\to }{CR}}^{W}$	${\overset{\to }{CR}}^{BESS}$	${\overset{\to }{CR}}^{MG}$
$i_{1_{d}}$ (A)	69.42	30.00	17.00	-12.00	34.42
$i_{1_{q}}$ (A)	-35.89	-6.53	-8.83	-20.51	0
$i_{2_{d}}$ (A)	-15.07	-2.47	-3.33	-9.27	0
$i_{2_{q}}$ (A)	-20.73	-3.46	-5.47	-11.80	0
$i_{0m_{d}}$ (A)	8.11	1.04	1.05	6.02	0
$i_{0m_{q}}$ (A)	3.58	-1.57	-1.31	6.46	0

**Figure 10:**
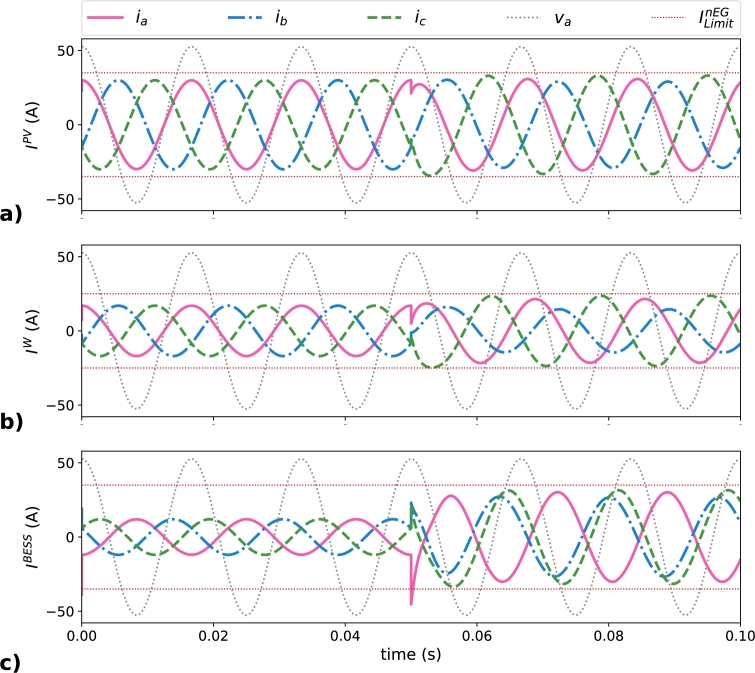
Instantaneous phase current in the EGs in the optimal distribution scenario: a) ***I***^*PV*^, b) ***I***^*W*^, and c) ***I***^*BESS*^.

Instantaneous currents in the EGs for the optimal distribution approach are plotted in Fig. 10; where Fig. 10a corresponds to $I^{PV}$, Fig. 10b corresponds to $I^{W}$, and Fig. 10c corresponds to $I^{BESS}$. The $I_{Limit}^{nEG}$ is included in the plots as two horizontal dashed lines. The peak value of each phase current is shown in Table 7. The solution obtained shows that all $I^{nEG}$ are smaller than theirs $I_{Limit}^{nEG}$, optimizing the distribution of the fundamental non-efficient load current terms between the EGs present in the MG. Phase *a* of the PV generator presents a current of 30.8 A, smaller than the maximum of 35 A available for this EG. The difference in the BESS is smaller, with 31.5 A in phase *c* with respect to the maximum of 35 A. The smallest margin is seen in the wind generator, in which the current of phase *c* is 23.6 A when the maximum for this EG is 25 A.

**Table 7:** Peak phase currents (in A) in the EGs in the optimal distribution scenario.

MG element	Phase current (A)	*t*<0.05 s	*t*>0.05 s
	${I_{a}}^{PV}$	30	30.82
***I***^*PV*^	${I_{b}}^{PV}$	30	29.11
(target=35 A)	${I_{c}}^{PV}$	30	33.11

	${I_{a}}^{W}$	17	21.46
***I***^*W*^	${I_{b}}^{W}$	17	14.63
(target=25 A)	${I_{c}}^{W}$	17	23.63

	${I_{a}}^{BESS}$	-12	30.02
***I***^*BESS*^	${I_{b}}^{BESS}$	-12	26.60
(target=35 A)	${I_{c}}^{BESS}$	-12	31.56

Regarding the proposed optimal solution, the optimization method guarantees EGs limit even if the ${{\overset{\to }{CR}}^{Load}}_{N-eff}$ terms are bigger than EGs capacities because the minimization in this optimization problem looks for ${{\overset{\to }{CR}}^{MG}}_{N-eff}$ equal to zero. However, if the target is not reached, the result guarantees the minimum values for ${{\overset{\to }{CR}}^{MG}}_{N-eff}$. It can be concluded that in this scenario ${{\overset{\to }{CR}}^{Load}}_{N-eff}$ distribution among EGs was the best of all analyzed in this paper.

## 5 Discussion

Four scenarios have been simulated to demonstrate the validity of the SoCR proposal and the optimization procedure developed to distribute ${{\overset{\to }{CR}}^{Load}}_{N-eff}$ between the EGs connected in an MG, trying to improve the power quality in the MG and reaching the operating condition in which the non-efficient current terms in the MG are null (${{\overset{\to }{CR}}^{MG}}_{N-eff}=0$). Characteristics of the linear unbalanced load are kept constant in the four scenarios, varying the operating conditions of the EGs present in the MG, that includes a PV, a wind generator, and a BESS. The maximum instantaneous current targets at the output of the PV and wind generator are set at 35 A and 25 A respectively. The BESS can supply or consume fundamental positive-sequence active power at the same time that provide ${{\overset{\to }{CR}}^{Load}}_{N-eff}$ to the MG, with a maximum instantaneous output current target equal to 35 A. Limits are imposed on the maximum instantaneous currents of the EGs to study the effect of current distribution under different operating conditions of an MG. Not considering the maximum ratings of EGs is a problem in an MG because exceeding the limit can cause the EG to shut down, which implies a loss of the renewable energy source or the BESS, and the circulation of non-efficient currents through the MG (${{\overset{\to }{CR}}^{MG}}_{N-eff}\neq 0$). Table 8 is a summary of peak currents in the EGs for the four presented scenarios.

**Table 8:** Summary of peak currents (in A) in the EGs in the four presented scenarios.

MG element	phase	Baseline scenario	Balanced distribution scenario	Proportional distribution scenario	Optimal distribution scenario
	${I_{a}}^{PV}$	0	32.33	27.55	30.82
***I***^*PV*^	${I_{b}}^{PV}$	0	24.29	29.95	29.11
(target=35 A)	${I_{c}}^{PV}$	0	**41.32**	34.08	33.11

	${I_{a}}^{W}$	0	22.95	**25.13**	21.46
***I***^*W*^	${I_{b}}^{W}$	0	11.42	13.05	14.63
(target=25 A)	${I_{c}}^{W}$	0	**29.63**	19.64	23.63

	${I_{a}}^{BESS}$	**-75.16**	-22.75	**-37.59**	30.02
***I***^*BESS*^	${I_{b}}^{BESS}$	**-59.64**	-18.00	-23.52	26.60
(target=35 A)	${I_{c}}^{BESS}$	-18.86	-16.15	-30.09	31.56

The baseline scenario analyzed first only uses the BESS and the load to verify the suitability of the SoCR to correctly represent the load current terms (${\overset{\to }{CR}}^{Load}$) and study the case where a single EG is in charge of compensating all ${{\overset{\to }{CR}}^{Load}}_{N-eff}$, such as when an SAPF is installed in the MG. Under these operating conditions in the MG, the current target of the BESS is largely exceeded in phases *a* and *b* when the load is connected, as highlighted by the bold values included in Table 8 for the baseline scenario column. This case in which only one EG compensates all ${{\overset{\to }{CR}}^{Load}}_{N-eff}$ requires a very oversized EG when other existing EGs in the MG could collaborate in improving the operating conditions of the MG.

The second approach proposes a balanced distribution of the fundamental non-efficient load current terms considering all the EGs in the MG, which in the case studied corresponds to one third of ${{\overset{\to }{CR}}^{Load}}_{N-eff}$ for each EG. As is shown in Table 8, the peak phase currents in the BESS decrease in the balanced distribution scenario with respect to the baseline scenario, although PV and wind overcome $I_{Limit}^{EG}$ as it is presented in bold in the values given for phase *c* of these EGs. Although the distribution of ${{\overset{\to }{CR}}^{Load}}_{N-eff}$ improves between the EGs with the balanced distribution, the problem of exceeding the maximum current targets of the EGs remains.

The proportional distribution scenario assigns ${{\overset{\to }{CR}}^{Load}}_{N-eff}$ among EGs based on their remaining capacity depending on $i_{1_{d}}$ and $I_{Limit}^{EG}$. The proportional distribution has better behavior than both previous scenarios; however, current in phase *a* in wind and BESS exceed their corresponding $I_{Limit}^{EG}$, as it is shown in bold in the fifth column of Table 8. Although the proportional distribution scenario does not achieve that all EGs work within the targets set for their corresponding peak current, it is important to highlight that phase *a* of $I^{PV}$ has still some remaining capacity, which suggests that wind and BESS currents in phase *a* could be reduced to be within their nominal values.

An improvement of the point of operation of the MG is achieved in the optimal distribution scenario, being based on the optimization model presented in Equation (20). This scenario looks for ${{\overset{\to }{CR}}^{MG}}_{N-eff}$ minimizing, considering EGs limits as constraints and ensuring that $I_{Limit}^{EG}$ are not exceeded, as shown in Table 8. If the EG remaining capacities do not allow completely supplying ${{\overset{\to }{CR}}^{Load}}_{N-eff}$, the ${{\overset{\to }{CR}}^{MG}}_{N-eff}$ would be different to zero while the MG continues in operation. The optimal distribution scenario is the preferred compensation approach because it has the best ${{\overset{\to }{CR}}^{Load}}_{N-eff}$ distribution, can be implemented in autonomous form in an MG using an MGCC, permits to operate EGs without exceeding their current limits, and adapts to the instantaneous operating conditions present in the MG.

The simulation of the proposed scenarios leads to conclude that SoCR sets a benchmark for distribution of ${{\overset{\to }{CR}}^{Load}}_{N-eff}$ among EGs improving the power quality in the MG. The use of EGs as distributed power active filters avoid the installation of an active power filter only focused on the compensation of the non-efficient load current terms. The SoCR procedure also shown that ${{\overset{\to }{CR}}^{Load}}_{N-eff}$ distribution between the EGs can increase the equipment usage in MGs. For instance, PV is only used for part of the day, and with our proposal it could be in operation during the whole day. SoCR could be applied in commercial EGs, facilitating the adoption of MGs and leading to significant economic and environmental cost reductions in electric power systems by making better use of the capabilities of existing equipment.

## 6 Conclusion

An optimization model for non-efficient current distribution in a grid-tied MG has been developed and simulated in a small size MG considering only fundamental positive-sequence voltages. The grid-tied MG is composed of three EGs and a linear unbalanced load. The non-efficient load current distribution uses a system of six constant reference terms denoted as SoCR, being applied in this work to improve power quality in the MG. SoCR is based on symmetrical components and *dq* transformation allowing representing a linear unbalanced system and decoupling fundamental active current from fundamental non-efficient current terms. After application of SoCR to the load currents, fundamental non-efficient current terms can be distributed between EGs without affecting the renewable resources, in which EGs current limit are considered. A new method of calculating the peak currents in each phase is proposed in the article. The approach avoids the use of square roots in the calculation of the output currents (generation + compensation) that each EG must provide. In this way the optimization algorithm can determine the currents of the EGs considering their operating limits without the non-convex problems that appears during the calculations of the optimization algorithm.

The SoCR distribution was applied in an MG throughout an MGCC which distributes the non-efficient current terms. Although this paper presents an optimization model that attempts to find the best distribution of the fundamental non-efficient load current terms between EGS, three previous scenarios were analyzed to show the power quality improvement in MGs when considering the current capabilities of the EGs under different SoCR approaches. However, if the current in one of the phases of any EG is exceeded, the corresponding EG could be disconnected by circuit breakers that affect renewable resources.

Four different scenarios for the distribution of compensation are simulated to show SoCR behavior: baseline, balanced, proportional, and optimal. In those scenarios, all SoCR of the fundamental non-efficient load current terms are obtained and used as part of the current references for PV, wind, and BESS in different distribution scenarios of the compensation of the non-efficient terms.

Baseline scenario is used for SoCR methodology validation, with the BESS operating as an SAPF (compensation of all the fundamental non-efficient load current terms) at the same time that supply fundamental positive-sequence active current to the MG (operating as a source of fundamental positive-sequence active power). This scenario allows showing the decoupling phenomena among fundamental positive-sequence active current and ${{\overset{\to }{CR}}^{Load}}_{N-eff}$. Although this scenario enhances power quality in the MG, the $I_{Limit}^{BESS}$ target was overcome, so if only one EG is used for providing non-efficient load current terms, the EG size would increase.

The three EGs present in the MG are used in the second scenario, in which a balanced distribution of ${{\overset{\to }{CR}}^{Load}}_{N-eff}$ between the EGs is proposed. Despite this distribution enhances power quality in the MG and performs the compensation using all EGs, target $I_{Limit}^{nEG}$ are exceeded.

The proportional distribution scenario considers the current remaining capabilities of the EGs, calculated as the target $I_{Limit}^{nEG}$ minus the fundamental positive-sequence active current. ${{\overset{\to }{CR}}^{Load}}_{N-eff}$ are distributed considering the remaining current of the EGs, obtaining an important reduction in the phase currents of the EGs. Regardless of this benefit, target $I_{Limit}^{nEG}$ are also exceeded in this scenario and the MG is not operating in the best operating conditions.

The contribution of this article to the improvement of the performance of the MG consists of the use of an optimization model for the distribution of ${{\overset{\to }{CR}}^{Load}}_{N-eff}$ between the EGs present in the MG. The optimal distribution scenario considers EG limits as constraints, and achieves ${{\overset{\to }{CR}}^{MG}}_{N-eff}=0$ better than the other distribution scenarios analyzed in this work and keeping the EGs in smaller rated values than the other scenarios. For those reasons, optimal distribution scenario is the best presented distribution because the optimal scenario was the only one that did not exceed the limits. Moreover, it is important to remark that all simulation enhanced power quality conditions in the MG, it means that after non-efficient distribution using SoCR methodology, there are not reactive and unbalance currents upstream MG.

The proposed use of SoCR methodology and the optimization model for distribution of non-efficient current terms reduce cost and amount of elements for manufacturing equipment; also, if SoCR for non-efficient current distribution is implemented in commercial EGs, this methodology potentializes the energy transition in MG implementation.

## Funding statement

This work was supported by the Bicentenary Doctoral Excellence Scholarship Program, Court 1, with agreement number 20230017-20-20, under the call of the Science, Technology and Innovation Fund of the General System of Royalties (High Level Training Project Universidad de Antioquia National BPIN 2019000100017).

## CRediT authorship contribution statement

Benavides-Córdoba Santiago, Urrea-Quintero Jorge-Humberto, Muñoz-Galeano Nicolás, Cano-Quintero Juan-B, and Segui-Chilet Salvador: Conceived and designed the experiments; Performed the experiments; Analyzed and interpreted the data; Contributed reagents, materials, analysis tools or data; Wrote the paper.

## Declaration of Competing Interest

The authors declare that they have no known competing financial interests or personal relationships that could have appeared to influence the work reported in this paper.

## Data Availability

Data included in article/supp. material/referenced in article.
